# CDC-WONDER database analysis of external causes of mortality and morbidity in the United States

**DOI:** 10.1097/MD.0000000000048448

**Published:** 2026-04-24

**Authors:** Hana Zhu, Junsheng Jiang

**Affiliations:** a Department of Pediatrics, Zhejiang Chinese Medical University, Hangzhou, Zhejiang, China; b Department of Pediatrics, The First Affiliated Hospital of Yuhang District, Hangzhou, Zhejiang, China; c Department of Pediatrics, The First People’s Hospital of Linping District, Hangzhou, Zhejiang, China.

**Keywords:** age-adjusted mortality rates, CDC WONDER, external cause

## Abstract

External causes of mortality, such as accidents, suicide, and homicide, remain a significant public health concern in the United States. Although previous studies have examined injury-related mortality in specific populations or over shorter timeframes, comprehensive analyses of long-term national trends across demographic subgroups are limited. This study analyzed trends in external cause-related deaths and age-adjusted mortality rates (AAMR) in the U.S. (1999–2023) and subgroup differences to improve the understanding of long-term patterns and identify populations that may benefit from future targeted interventions. Data were extracted from the CDC WONDER Database. Key indicators included death counts, percent change in deaths, AAMR (with 95% confidence interval [CI]), and average annual percent change (AAPC, with 95% CI). Stratified analyses were performed by sex, census region, race/ethnicity, urbanization level, and age group. The Joinpoint Regression Program was used to determine trends in mortality within the study period. Joinpoint regression analysis was employed to determine annual percentage changes (APCs) and assess statistical significance (*P* < .05). A total of 1,234,567 external cause deaths were recorded between 1999 and 2023, with males accounting for approximately 73% of all deaths. Total external cause deaths rose by 5.77% (1999: 29,642; 2023: 31,353), while AAMR slightly declined (1999: 30.08 [29.73–30.42]; 2023: 28.85 [28.53–29.17]) with a non-significant AAPC (0.13 [−0.96–1.23]). Male deaths rose more (6.33%) than females (4.19%), with higher male AAMR (both AAPCs non-significant). Deaths fell in the Northeast (−16.22%) and Midwest (−4.32%), rose in the South (16.00%) and West (9.80%); only the Northeast had significant downward AAMR (AAPC: −0.55 [−0.88–−0.21], *P* < .05). Deaths increased most in non-Hispanic (NH) Other (84.54%) and Hispanic (65.83%) groups, fell in NH White (−23.58%); NH Black AAMR rose (1999: 46.30; 2023: 57.45, AAPC non-significant). The “NH Other” category includes individuals identifying as American Indian or Alaska Native, Asian, Native Hawaiian, or Other Pacific Islander, as classified by the CDC WONDER database. Metropolitan deaths rose (15.37%), non-metropolitan deaths fell (−28.34%); non-metropolitan areas had significant downward AAMR (AAPC: −0.67 [−1.26–−0.07], *P* < .05). Deaths rose in infants aged < 1 year (30.87%) and individuals aged 15 to 24 years (14.43%), fell in children aged 1 to 4 years (−32.14%) and children aged 5 to 14 years (−31.30%); the 1 to 4 and 5 to 14 years age groups had significant downward crude mortality (AAPCs: −1.15, −1.50, *P* < .05). Total U.S. external cause deaths slightly increased, with stable overall AAMR, but significant subgroup differences highlight the need for targeted interventions.

## 1. Introduction

In recent years, trends in mortality and morbidity due to external causes (including accidents, suicide, homicide, etc) in the United States have become an important topic in public health research.^[1,2]^ According to existing studies, mortality rates from external causes vary significantly across different populations and regions, and are closely associated with socioeconomic factors, accessibility of healthcare resources, and prevention policies.^[3,4]^ For example, research by Case and Deaton found that between 1999 and 2013, mortality rates among middle-aged NH white Americans increased significantly due to drug overdoses, suicide, and alcohol-related diseases – a raise trend not observed in other developed countries.^[3]^ Additionally, standardized frameworks for injury mortality data (such as the E-code matrix recommended by the CDC) provide a basis for cross-regional and cross-population comparisons.^[1]^

However, most existing studies focus on specific populations (such as children, adolescents, or racial/ethnic groups) or shorter time spans, lacking a comprehensive analysis of recent nationwide trends, particularly in-depth exploration of urban-rural disparities, racial/ethnic stratification, and age-specific variations.^[5–7]^ This study aims to use the CDC WONDER database to analyze trends in U.S. mortality rates from external causes between 1999 and 2023, and apply Joinpoint regression models to assess the annual percentage change (APC), thereby revealing dynamic shifts across different sexs, census regions, racial/ethnic groups, urbanization levels, and age groups, and to provide evidence for targeted prevention strategies.

## 2. Methods

### 2.1. Study participants

This study extracted de-identified, publicly available mortality data from the CDC WONDER (Wide-ranging Online Data for Epidemiologic Research) database, a comprehensive resource maintained by the U.S. Centers for Disease Control and Prevention (CDC) National Center for Health Statistics that compiles data from the U.S. National Vital Statistics System, and no ethics approval or informed consent was required for this secondary data analysis; data were retrieved for all deaths occurring in the United States from 1999 to 2023 where external causes were listed as either the underlying or contributing cause on death certificates, with external cause-related mortality cases identified using International Classification of Diseases, 10th Revision (ICD-10) codes V01-Y09 in accordance with CDC guidelines for national mortality research,^[8]^ and while these codes are the gold standard for population-level surveillance, potential misclassification bias may exist due to the documentation of multiple causes of death on individual death certificates.

### 2.2. Outcomes

The primary study outcomes included absolute counts of external cause-related deaths for each study year and stratified subgroup, the percent change in death counts between the baseline year 1999 and 2023, crude mortality rates and age-adjusted mortality rates (AAMR) with 95% CIs for external causes of mortality, as well as APC and average annual percent change (AAPC) with 95% CI to characterize temporal trends in mortality rates, with crude mortality rates used in place of AAMR for age group analyses due to narrow age stratification eliminating the need for age adjustment, and 2020 AAMR data substituted for 2023 AAMR data in urbanization level analyses due to CDC WONDER database availability constraints, with AAPC thus calculated for the 1999 to 2020 period for this subgroup.

### 2.3. Covariates

Relevant covariates extracted from the CDC WONDER database and defined based on official U.S. national classification frameworks included sex, race/ethnicity, age groups, urban–rural classification, U.S. census region and U.S. state, with sex classified as male or female, race/ethnicity divided into 4 mutually exclusive CDC-defined groups (NH White, NH Black, Hispanic, and NH Other, the latter including American Indian or Alaska Native, Asian, Native Hawaiian, or Other Pacific Islander individuals), age stratified into 4 pediatric and young adult groups (<1 year, 1–4 years, 5–14 years, 15–24 years), urban–rural classification determined by the 2013 NCHS Urban–Rural Classification Scheme for U.S. Counties (applied consistently across the study period) which divides areas into metropolitan (large: ≥1 million population; medium/small: 50,000 to 999,999 population) and non-metropolitan (rural: <50,000 population) regions, and U.S. census region categorized into Northeast, Midwest, South, and West per the United States Census Bureau definitions, with all covariate stratification performed for the United States at the census region, state, and sub-demographic levels.^[9,10]^

### 2.4. Statistical analysis

Crude mortality rates were computed as the number of external cause-related deaths per 100,000 corresponding U.S. population, while AAMRs were standardized to the 2000 United States standard population – the widely used fixed age-distributed reference population for U.S. national mortality research – via the direct standardization method, and temporal trends in AAMRs (and crude mortality rates for age group analyses) were analyzed using the Joinpoint Regression Program, with the number of statistically significant joinpoints determined via the Monte Carlo permutation method, 95% CIs for AAMRs calculated using standard error estimates from the program, and APCs calculated for individual time segments and AAPCs as the weighted average of APCs for the overall study period, both reported with 95% CIs; a log-linear model and weighted least squares were applied for joinpoint regression analyses, trends were considered statistically significant if APC or AAPC differed from zero with statistical significance set at *P* ≤ .05, and exact *P*-values were used to indicate significance rather than only asterisks, with stratified analyses conducted for all covariates to quantify disparities in external cause mortality across subgroups, and clinical trial registration deemed not applicable for this retrospective secondary data analysis

## 3. Results

### 3.1. Overall trends in external cause deaths and AAMR

**Figure 1.**
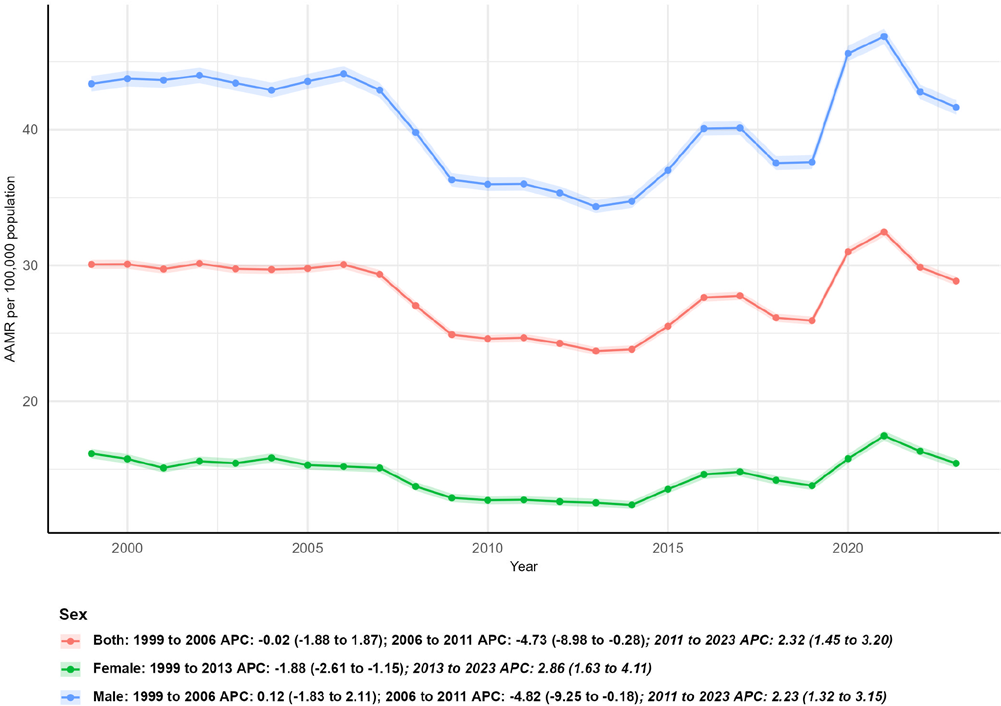
Overall trends in external cause deaths and AAMR. AAMR = age-adjusted mortality rate.

From 1999 to 2023, the total number of deaths due to external causes in the United States increased by 5.77%, rising from 29,642 cases in 1999 to 31,353 cases in 2023. In contrast, the age-adjusted mortality rate (AAMR) showed a slight downward trend over the same period: the AAMR was 30.08 per 100,000 population (95% CI: 29.73–30.42) in 1999 and decreased to 28.85 per 100,000 population (95% CI: 28.53–29.17) in 2023. The AAPC of AAMR was 0.13 (95% CI: −0.96–1.23), which was not statistically significant (*P* > .05) (Table 1 and Fig. 1).

**Table 1:** External causes deaths and AAMR in the United States from 1999 to 2023 and their changing trends.

Characteristic	Deaths			AAMR		
	1999	2023	Percent change (%)	1999 (95% CI)	2023 (95% CI)	AAPC (95% CI)
Total	29,642	31,353	5.77	30.08 (29.73–30.42)	28.85 (28.53–29.17)	0.13 (−0.96–1.23)
Sex						
Female	7756	8081	4.19	16.15 (15.79–16.51)	15.43 (15.09–15.77)	0.07 (−0.56–0.69)
Male	21,886	23,272	6.33	43.37 (42.79–43.94)	41.63 (41.10–42.17)	0.11 (−1.03–1.26)
Census region						
Northeast	3816	3197	−16.22	21.79 (21.10–22.49)	18.05 (17.42–18.68)	−0.55 (−0.88–−0.21)*
Midwest	6948	6648	−4.32	30.42 (29.71–31.14)	29.43 (28.72–30.14)	0.20 (−0.39–0.80)
South	12,585	14,598	16.00	35.65 (35.02–36.27)	34.17 (33.62–34.73)	0.09 (−0.91–1.09)
West	6293	6910	9.80	27.57 (26.89–28.25)	26.79 (26.16–27.42)	−0.12 (−1.54–1.32)
Race						
Hispanic	4501	7464	65.83	27.50 (26.70–28.31)	27.12 (26.51–27.74)	−0.10 (−1.20–1.01)
NH Black	6686	8557	27.98	46.30 (45.19–47.41)	57.45 (56.23–58.67)	0.82 (−0.75–2.41)
NH White	17,169	13,120	−23.58	27.45 (27.04–27.86)	24.02 (23.60–24.43)	−0.21 (−1.76–1.36)
NH other	1177	2172	84.54	22.48 (21.19–23.76)	18.81 (18.02–19.60)	−0.44 (−1.56–0.68)
Urbanization*						
Metropolitan	23,132	26,688	15.37	27.85 (27.49–28.21)	30.02 (29.66–30.37)	0.04 (−1.10–1.18)
Nonmetropolitan	6510	4665	−28.34	41.79 (40.77–42.81)	37.43 (36.43–38.43)	−0.67 (−1.26–−0.07)*
Age groups						
<1 yr	1176	1539	30.87	30.98 (29.21–32.75)	42.18 (40.07–44.29)	1.47 (−0.02–2.99)
1–4 yr	2265	1537	−32.14	14.77 (14.16–15.37)	10.34 (9.82–10.86)	−1.15 (−1.62–−0.68)*
5–14 yr	3729	2562	−31.30	9.14 (8.84–9.43)	6.25 (6.01–6.49)	−1.50 (−2.80–−0.19)*
15–24 yr	22,472	25,715	14.43	58.10 (57.34–58.86)	58.59 (57.88–59.31)	0.38 (−0.91–1.69)

AAMR = age-adjusted mortality rate, AAPC = average annual percent change, CI = confidence interval, NH = non-Hispanic.

* *P* < .05 (all unmarked results indicate *P* > .05).

### 3.2. Subgroup analysis by sex

**Figure 2.**
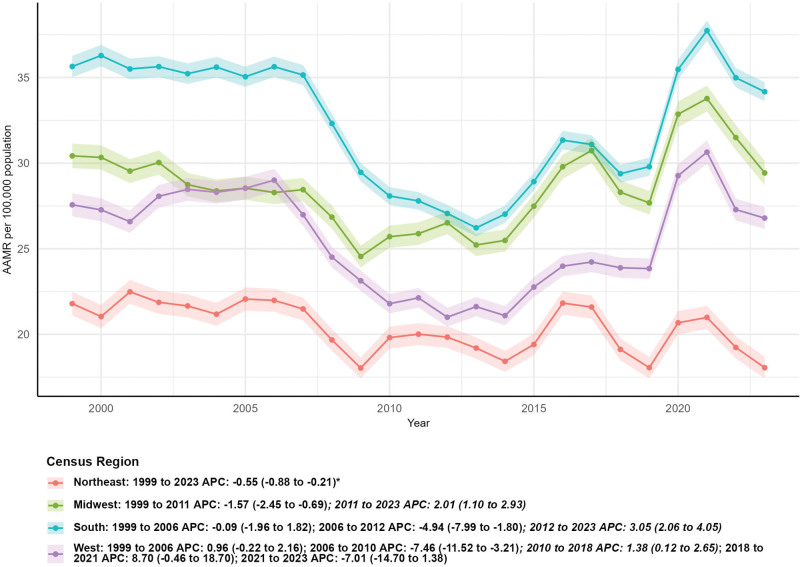
Subgroup analysis by sex.

In terms of sex stratification, the number of external cause deaths increased in both males and females, but the growth rate was higher in males. Male deaths rose by 6.33% (from 21,886 in 1999 to 23,272 in 2023), while female deaths increased by 4.19% (from 7756 in 1999 to 8081 in 2023). Consistently, male AAMR remained much higher than female AAMR throughout the study period: male AAMR decreased from 43.37 per 100,000 population (95% CI: 42.79–43.94) in 1999 to 41.63 per 100,000 population (95% CI: 41.10–42.17) in 2023, with an AAPC of 0.11 (95% CI: −1.03–1.26); female AAMR declined from 16.15 per 100,000 population (95% CI: 15.79–16.51) in 1999 to 15.43 per 100,000 population (95% CI: 15.09–15.77) in 2023, with an AAPC of 0.07 (95% CI: −0.56–0.69). Neither male nor female AAMR showed a statistically significant trend (*P* > .05) (Fig. 2).

### 3.3. Subgroup analysis by regional

**Figure 3.**
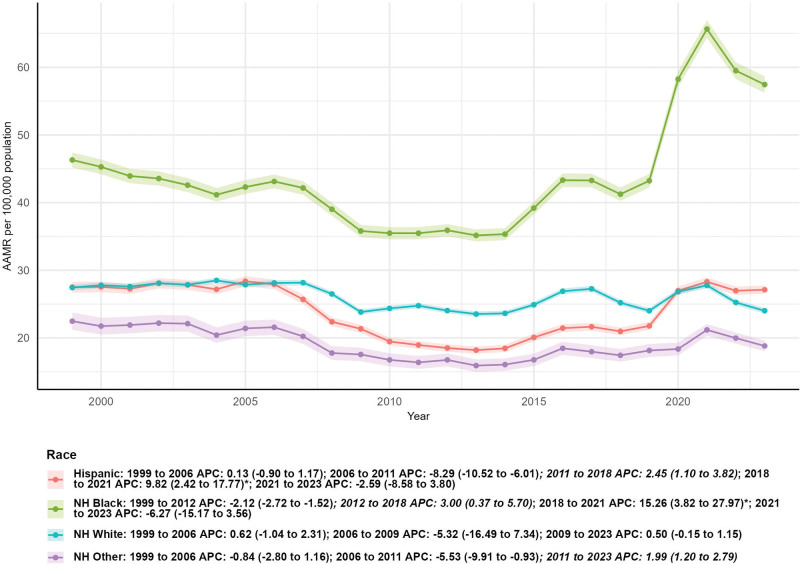
Subgroup analysis by regional.

Regional differences in external cause mortality were notable. The number of deaths decreased in the Northeast and Midwest regions: by 16.22% (from 3816 in 1999 to 3197 in 2023) in the Northeast and by 4.32% (from 6948 in 1999 to 6648 in 2023) in the Midwest. In contrast, deaths increased in the South and West: by 16.00% (from 12,585 in 1999 to 14,598 in 2023) in the South and by 9.80% (from 6293 in 1999 to 6910 in 2023) in the West. For AAMR trends, only the Northeast region showed a statistically significant downward trend, with an AAPC of −0.55 (95% CI: −0.88–−0.21, *P* < .05). The Midwest had a non-significantupward AAPC of 0.20 (95% CI: −0.39–0.80), the South had a non-significant upward AAPC of 0.09 (95% CI: −0.91–1.09), and the West had a non-significant downward AAPC of −0.12 (95% CI: −1.54–1.32) (*P* > .05 for all 3 regions) (Fig. 3, Fig. 1).

### 3.4. Subgroup analysis by race/ethnicity

**Figure 4.**
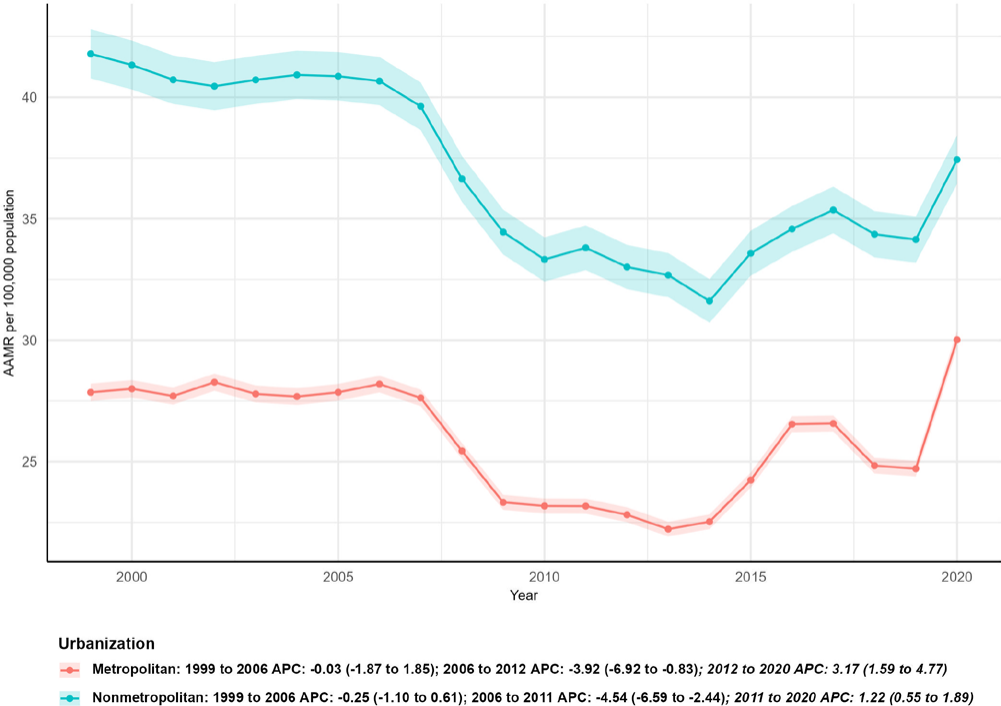
Subgroup analysis by race/ethnicity.

Large variations in mortality trends were observed across racial/ethnic groups (Fig. 4: race/ethnicity). The number of external cause deaths increased most substantially in the non-Hispanic (NH) Other group (84.54%, from 1177 in 1999 to 2172 in 2023) and the Hispanic group (65.83%, from 4501 in 1999 to 7464 in 2023), followed by the NH Black group (27.98%, from 6686 in 1999 to 8557 in 2023). Only the NH White group experienced a decrease in deaths, with a 23.58% reduction (from 17,169 in 1999 to 13,120 in 2023). In terms of AAMR, the NH Black group showed an upward trend in AAMR (from 46.30 per 100,000 population [95% CI: 45.19–47.41] in 1999 to 57.45 per 100,000 population [95% CI: 56.23–58.67] in 2023), but the AAPC of 0.82 (95% CI: −0.75–2.41) was not statistically significant. The NH White group’s AAMR decreased from 27.45 per 100,000 population (95% CI: 27.04–27.86) in 1999 to 24.02 per 100,000 population (95% CI: 23.60–24.43) in 2023, with a non-significant AAPC of −0.21 (95% CI: −1.76–1.36). The Hispanic group’s AAMR remained relatively stable (1999: 27.50 per 100,000 [95% CI: 26.70–28.31]; 2023: 27.12 per 100,000 [95% CI: 26.51–27.74]), with an AAPC of −0.10 (95% CI: −1.20–1.01). The NH Other group’s AAMR decreased from 22.48 per 100,000 population (95% CI: 21.19–23.76) in 1999 to 18.81 per 100,000 population (95% CI: 18.02–19.60) in 2023, with a non-significant AAPC of −0.44 (95% CI: −1.56–0.68) (*P* > .05 for all racial/ethnic groups).

### 3.5. Subgroup analysis by urbanization level

**Figure 5.**
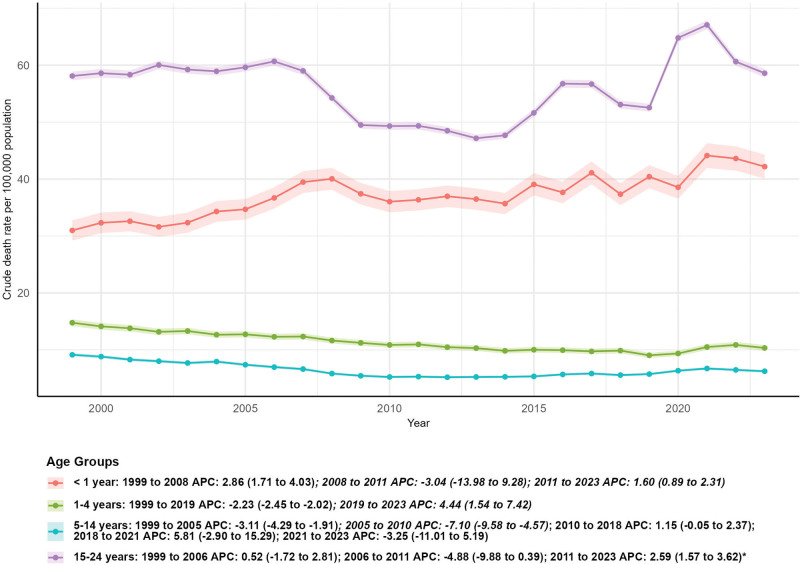
Subgroup analysis by urbanization level.

Due to data availability, 2020 AAMR data was used as a substitute for 2023 AAMR in the urbanization analysis, and AAPC was calculated for the 1999 to 2020 period. The number of external cause deaths increased by 15.37% in metropolitan areas (from 23,132 in 1999 to 26,688 in 2023) but decreased by 28.34% in non-metropolitan areas (from 6510 in 1999 to 4665 in 2023). For AAMR trends, non-metropolitan areas exhibited a statistically significant downward trend, with an AAPC of −0.67 (95% CI: −1.26–−0.07, *P* < .05). In contrast, metropolitan areas had a non-significant upward AAPC of 0.04 (95% CI: −1.10–1.18, *P* > .05) (Fig. 5).

### 3.6. Subgroup analysis by age group

**Figure 6.**
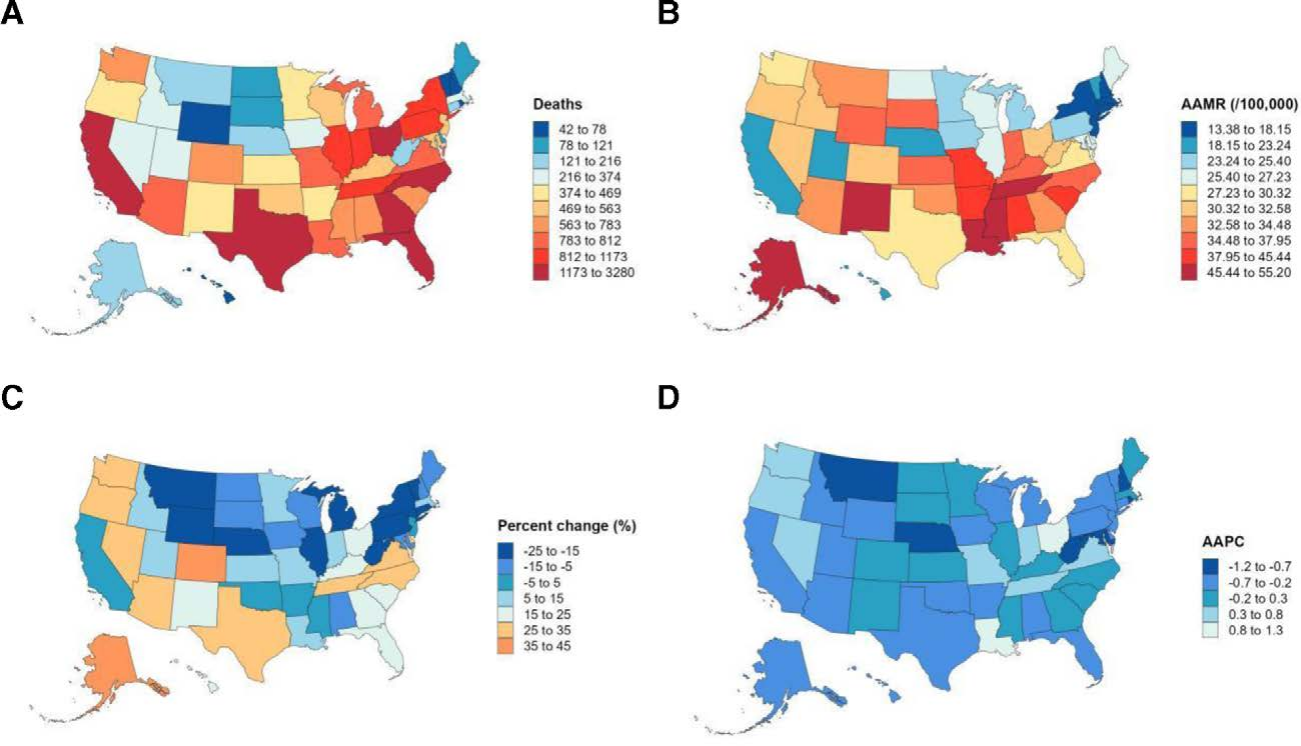
Subgroup analysis by age group.

Crude mortality rate was used instead of AAMR for age group analysis, and AAPC was computed based on crude mortality rates. The number of external cause deaths increased in infants aged < 1 year (30.87%, from 1176 in 1999 to 1539 in 2023) and individuals aged 15 to 24 years (14.43%, from 22,472 in 1999 to 25,715 in 2023), while decreasing in children aged 1 to 4 years (32.14%, from 2265 in 1999 to 1537 in 2023) and children aged 5 to 14 years (31.30%, from 3729 in 1999 to 2562 in 2023). Statistically significant downward trends in crude mortality rate were observed in children aged 1 to 4 years (AAPC: −1.15, 95% CI: −1.62–−0.68, *P* < .05) and children aged 5 to 14 years (AAPC: −1.50, 95% CI: −2.80–−0.19, *P* < .05). Infants aged < 1 year had a non-significant upward AAPC of 1.47 (95% CI: −0.02–2.99), and individuals aged 15 to 24 years had a non-significant upward AAPC of 0.38 (95% CI: −0.91–1.69) (*P* > .05 for both groups) (Fig. 6).

## 4. Discussion

This study aimed to analyze trends in external cause-related deaths and AAMR in the United States from 1999 to 2023, and explore disparities in these trends across key demographic, geographic and urbanization subgroups, with the ultimate goal of identifying high-risk populations to inform targeted public health interventions. The major findings of this study include that total U.S. external cause deaths increased by 5.77% over the study period while the overall AAMR showed a slight non-significant decline (AAPC = 0.13%); male external cause mortality remained consistently higher than that of females with a faster growth in death counts; external cause deaths decreased in the Northeast and Midwest but rose in the South and West, with only the Northeast showing a significant downward AAMR trend; NH Other and Hispanic groups saw the largest increases in death counts while NH White deaths declined, and NH Black AAMR exhibited an upward trend; metropolitan areas had a rise in external cause deaths while non-metropolitan areas saw a decrease with a significant AAMR decline; and external cause deaths increased in infants aged < 1 year and individuals aged 15 to 24 years but decreased in children aged 1 to 4 and 5 to 14 years, with the latter 2 age groups showing significant crude mortality declines. This study’s systematic analysis of 25 years of national external cause mortality data across multiple stratified subgroups fills a gap in existing research that often focuses on specific populations or shorter timeframes, and the findings provide population-specific evidence to guide the development of targeted external cause mortality prevention and intervention strategies in the U.S., with potential implications for public health practice both nationally and for other regions with similar demographic and geographic characteristics.

From an epidemiological perspective, the apparent paradox of a 5.77% increase in the total number of deaths alongside a decreasing trend in the AAMR (from 30.08 to 28.85 per 100,000) reflects the complex interplay between demographic shifts and public health interventions.^[11]^ Population aging is likely a key contributor to the rising death count,^[12]^ while the decline in AAMR indicates a genuine improvement in age-standardized mortality risk. This trend coincides with increased US investments in injury prevention over recent decades, including improvements in road traffic safety,^[13]^ enhanced workplace safety standards,^[14]^ and the implementation of violence prevention programs.^[15]^ However, the AAPC of 0.13% was small and did not reach statistical significance (*P* > .05), indicating that the effectiveness of current interventions requires further enhancement.^[16]^

Sex disparities show that mortality rates for males consistently exceeded those for females (2023 AAMR: Male 41.63/100,000 vs Female 15.43/100,000). This finding aligns strongly with theories of sex role socialization.^[17]^ Men are often overrepresented in high-risk occupations,^[18]^ more likely to engage in risk-taking behaviors,^[19]^ and demonstrate lower willingness to seek medical help.^[20]^ From a biopsychosocial model perspective, these differences reflect the profound impact of sex norms on health behaviors.^[21]^ Geographic analysis reveals uneven development across US regions. The significant decline in AAMR in the Northeast (AAPC = −0.55, *P* < .05) may be attributed to its more robust social safety nets, higher healthcare resource accessibility, and stricter public safety regulations.^[22,23]^ Conversely, a 16.00% increase in the number of deaths in the South highlights the region’s relatively weaker socioeconomic infrastructure, limited allocation of healthcare resources, and potential disparities in policy implementation.^[24,25]^ Disparities across racial/ethnic groups are particularly noteworthy. The notable increase in AAMR for the NH Black population (from 46.30 to 57.45 per 100,000) is closely linked to racial/ethnic disparities in healthcare access, socioeconomic status, and educational attainment.^[26,27]^ Structural racism likely impacts health outcomes through pathways such as restricted access to healthcare, poorer residential environmental quality, and unequal exposure to occupational risks.^[28,29]^ In contrast, the decline in AAMR for the NH White population (from 27.45 to 24.02 per 100,000) may reflect the cumulative effects of this group’s relative advantages in healthcare access and socioeconomic resources.^[30]^

Analysis by urbanization level indicates a significant decline in AAMR in nonmetropolitan areas (AAPC = −0.67, *P* < .05), potentially benefiting from advancements in telehealth technology, improvements in rural healthcare facilities, and targeted prevention programs.^[31,32]^ However, the 15.37% increase in the number of deaths in metropolitan areas underscores emerging challenges associated with urbanization, including increased population density, worsening traffic congestion, and social isolation.^[33]^ Age-specific patterns show significant mortality declines in the 1 to 4 and 5 to 14 year age groups, reflecting substantial achievements in child safety over the past 2 decades, such as increased use of child safety seats,^[34]^ improved toy safety standards, and the widespread implementation of school safety programs.^[35]^ Nonetheless, the 30.87% increase in infant deaths and the 14.43% increase in deaths among the 15 to 24 year age group are alarming. These trends may be linked to age-specific risk factors, such as infant sleep safety issues^[36]^ and increasing risky behaviors among adolescents and young adults,^[37]^ which are consistent with existing research highlighting the unique vulnerability of these age groups to external cause mortality and the need for age-tailored prevention strategies.

## 5. Study strengths

This study has several notable strengths that enhance the validity and value of its findings. First, it utilizes 25 years of comprehensive, population-based mortality data from the CDC WONDER database, a gold-standard public resource for U.S. mortality surveillance, ensuring the generalizability of results to the entire U.S. population. Second, the study conducts a rigorous stratified analysis across multiple key subgroups (sex, census region, race/ethnicity, urbanization level, age group), providing a detailed and nuanced understanding of external cause mortality disparities that is lacking in many existing studies focused on single populations or narrow geographic scopes. Third, the use of Joinpoint regression analysis allows for the precise quantification of temporal trends in mortality rates, including annual and AAPCs, with statistical significance testing, ensuring the robustness of the trend analysis. Finally, the study adheres to standardized CDC guidelines for identifying external cause deaths and calculating mortality rates, enhancing the comparability of its findings with other national and international public health research.

### 5.1. Study limitations and methodological considerations

This study has several limitations. First, the use of death certificate data may be subject to coding errors and misclassification biases. Second, the substitution of 2020 data for 2023 data in the urbanization analysis for 2023 might affect the accuracy of trend estimates. Third, while Joinpoint regression can identify trend changes, it cannot elucidate the underlying causal mechanisms. Finally, the failure to incorporate key social determinants of health, such as socioeconomic status and education level, limits a deeper understanding of health disparities.

## 6. Conclusion

Between 1999 and 2023, total external cause deaths in the U.S. saw a slight 5.77% increase with a small, non-significant decline in overall age-adjusted mortality rate, and significant disparities in mortality trends were observed across all demographic, geographic and urbanization subgroups analyzed. Males, residents of the Southern U.S., NH Black, NH Other and Hispanic groups, infants aged < 1 year and individuals aged 15 to 24 years emerged as key high-risk populations for rising external cause mortality, while the Northeast region, non-metropolitan areas, and children aged 1 to 4 and 5 to 14 years experienced reductions in external cause mortality or mortality rates. These findings confirm that external cause mortality in the U.S. is not a uniform public health issue and highlight the critical need for population-specific, targeted prevention and intervention strategies to address subgroup disparities. Strengthening cross-sector public health collaboration and integrating social determinant monitoring into mortality surveillance systems will be essential for reducing overall external cause mortality and advancing health equity in the U.S.

## Author contributions

**Conceptualization:** Hana Zhu.

**Data curation:** Hana Zhu.

**Project administration:** Junsheng Jiang.

**Resources:** Junsheng Jiang.

**Software:** Junsheng Jiang.

**Supervision:** Junsheng Jiang.

**Validation:** Junsheng Jiang.

**Visualization:** Junsheng Jiang.

**Writing – original draft:** Junsheng Jiang.

**Writing – review & editing:** Hana Zhu.
